# *Labisia pumila* var. *alata* Extract Induces Apoptosis Cell Death by Inhibiting the Activity of Oestrogen Receptors in MCF-7 Breast Cancer Cells

**DOI:** 10.3390/ijms26083748

**Published:** 2025-04-16

**Authors:** Muhammad Faiz Zulkifli, Zolkapli Eshak, Mohd Helmy Mokhtar, Wan Iryani Wan Ismail

**Affiliations:** 1 Cell Signaling and Biotechnology Research Group (CeSBTech), Faculty of Science and Marine Environment, Universiti Malaysia Terengganu, Kuala Nerus 21030, Terengganu, Malaysia; faizzulkifli90@yahoo.com; 2 Faculty of Pharmacy, Universiti Teknologi MARA, Selangor Branch, Puncak Alam Campus, Bandar Puncak Alam 42300, Selangor, Malaysia; zolkapli_eshak@uitm.edu.my; 3 Department of Physiology, Faculty of Medicine, Universiti Kebangsaan Malaysia, Kuala Lumpur 56000, Cheras, Malaysia

**Keywords:** *Labisia pumila* var. *alata*, MCF-7, oestrogenic activity, antiproliferative effect, biphasic effect, breast cancer, Malay traditional medicine

## Abstract

*Labisia pumila* var. *alata* (LP) is an herbaceous shrub commonly used by women to promote health and vitality, alleviate postmenopausal symptoms, and enhance libido. Research indicates that LP possesses significant oestrogenic and antiproliferative properties towards breast cancer; however, the specific mechanisms involved remain unclear. We investigate the oestrogenic effects of LP in inducing apoptosis in human breast adenocarcinoma (MCF-7) cells and the mechanisms underlying this process. Docking analysis reveals that the phytoestrogens in LP can bind to oestrogen receptors (ER), specifically ERα and ERβ. MTT assays demonstrate that LP has a dose- and time-dependent antiproliferative effect on MCF-7 cells. Furthermore, the antiproliferative activity of LP on MCF-7 cells is inhibited by Fulvestrant, indicating that its effects are mediated through oestrogen receptors. Flow cytometry analysis shows that the antiproliferative effect of LP results from the induction of apoptosis in MCF-7 cells. The activation of caspase 3, along with caspase 8 and caspase 9, suggests that LP triggers apoptosis through both intrinsic and extrinsic pathways. The findings regarding the aqueous extract of LP and its impact on the proliferative activity of MCF-7 cells may have significant therapeutic and preventive implications for future drug development, particularly in the context of breast cancer.

## 1. Introduction

Breast cancer is the most common cancer in women, accounting for 2.26 million (11.6%) new cases and 665,684 deaths globally in 2022 [1]. Breast cancer is second behind lung cancer (12.4%) as the most frequently detected cancer, followed by colorectal (9.6%), prostate (7.3%), and stomach cancer (4.9%) [1]. Similarly, breast cancer is also the most common cancer among Malaysian women, with 43,837 cases reported in 2018. Breast cancer tops the list with 17.3%, after colorectal (14%) and lung cancer (10.7%) [2]. Breast cancer has the third highest mortality rate at 11% after colorectal (12.9%) and lung cancer (15.4%) [2]. Chemotherapy, adjuvant hormonal therapy, and radiotherapy are used for patients with advanced or late-stage breast cancer. The antiproliferative effect of chemotherapeutic drugs is significant in proliferating cells, such as cancer cells. However, their toxicity to normal cells and tissues can have severe and irreversible consequences [3,4,5]. Therefore, it is essential to discover a safer and more natural approach that can serve as an adjunctive therapy or alternative to conventional treatments.

In breast cancer, a distinction is made between oestrogen-dependent and oestrogen-independent cancers, with the majority showing oestrogen-dependent cell proliferation by expressing high levels of oestrogen receptor (ER). Oestrogen regulates the cell cycle, apoptosis, and cell adhesion with or without activation of ER, which is essential for the survival of normal and malignant cells [6]. Furthermore, ER, especially ERα, is a prognostic factor in breast cancer cases.

Natural bioactive compounds, like phytoestrogens, have long been recognised as potent antioxidants and cancer-preventive agents. Many of the plant-based foods are rich in bioactive compounds that exhibit a range of multi-targeted activities against breast cancer cells, including antioxidant effects, the modulation of signalling pathways, and the induction of apoptosis (programmed cell death) [7,8,9,10,11,12]. Phytoestrogen is a plant-derived compound that induces biological responses by modulating or mimicking the action of oestrogens by binding to ERs [13]. This is because its chemical structure is similar to that of oestrogen; therefore, it may induce apoptosis by downregulating ER expression, which contributes to the treatment of breast cancer. Most phytoestrogens are phenolic compounds, of which isoflavones and coumestans are the most studied. Studies have shown that phytoestrogens, found in plants, fruits, leaves, and whole grains, can suppress the proliferation of breast cancer cells, acting as a significant strategy for primary prevention and helping to reduce the likelihood of developing the disease in the first place. Thus, many have incorporated phytoestrogen compounds as food additives to prevent chronic diseases [14,15,16,17,18].

*Labisia pumila* (LP), known in Malaysia as Kacip Fatimah, has been used as a health remedy for various diseases, especially for Malay women [19]. It is a small genus of plants from the Myrsinaceae family that is consumed as a decoction. The plant improves health conditions related to oestrogen imbalance and postpartum rejuvenation, relieves menstrual problems, and promotes sexual health [16,20,21]. Currently, the plant is marketed as a health supplement and tonic. To date, many studies have identified the phytochemical form of the LP plant with various beneficial pharmacological activities, including phenolics and flavonoids, β-carotene and ascorbic acid, and the derivatives of the alkenyl compound benzoquinone (Table 1) [11,19,21,22,23,24,25,26,27]. LP has also been reported to have antimicrobial and antifungal properties, exhibit skin protective properties when exposed to UV sunlight, and promote collagen synthesis [28,29,30]. In addition, LP has antiproliferative properties in skin cancer (HM3KO cell lines and skin carcinogenesis in mice), prostate cancer, colon cancer (HT-29 cell lines), and breast cancer (MCF-7 cell lines) [28,31]. However, the exact mechanism underlying LP’s antiproliferative effect is still being determined, and reports on the plant’s properties need to be further investigated.

Despite the growing interest in plant-derived compounds for cancer therapy, the specific oestrogenic potential, antiproliferative effects, and apoptotic mechanisms of LP in breast cancer cells remain poorly understood. Therefore, this study aims to investigate the oestrogenic and antiproliferative activities of LP, as well as the molecular mechanism of apoptosis induced by LP in human breast adenocarcinoma (MCF-7) cell lines, with the goal of identifying the signalling pathway involved.

## 2. Results

### 2.1. Molecular Docking Simulation of LP’s Phytoestrogens on Oestrogen Receptors

Molecular docking analysis was performed using AutoDock Vina software (version 1.2.0). Table 2 and Table 3 show the binding affinity (kcal/mol) and common amino acid value of the ligand-binding domains of ERα and ERβ involved in interactions with various phytochemicals in LP.

Molecular docking analysis reveals that all phytochemicals of the LP can bind to both receptors at the same binding site as 17β-oestradiol, with estimated binding affinities of −8.03 and −8.08 for all ligands in ERα and ERβ, respectively (Table 2). For molecular docking, a negative estimated binding affinity score indicates a favourable interaction, with more negative values representing stronger binding affinity. 17β-oestradiol showed the lowest binding affinity score at −9.6 and −11.2 for ERα and Erβ, respectively. Among the ligands, daidzein exhibited the strongest binding affinity score of −9.1 kcal/mol in both ERα and ERβ, followed by genistein with a score of −9.0 kcal/mol. In contrast, gallic acid showed the weakest binding affinity scores of −5.6 and −5.9 kcal/mol in ERα and ERβ, respectively.

**Figure 1:**
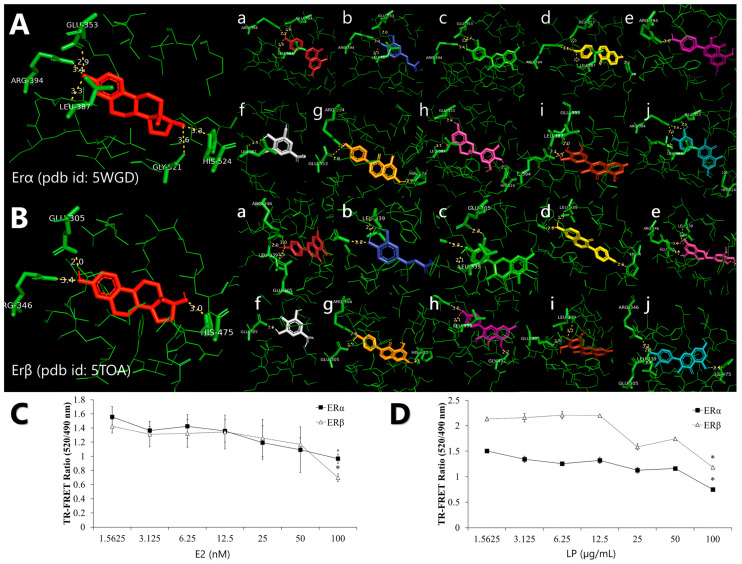
Representation of the binding interaction of *Labisia pumila* var. *alata* (LP) phytochemicals with (**A**) oestrogen receptor alpha (ERα) (PDB ID: 5WGD) and (**B**) oestrogen receptor beta (ERβ) (PDB ID: 5TOA), which was previously re-docked with 17β-oestradiol. All phytochemicals were compared with 17β-oestradiol, which is in red. Alphabetical order, (**a**) apigenin, (**b**) caffeic acid, (**c**) catechin, (**d**) daidzein, (**e**) epigallocatechin, (**f**) gallic acid, (**g**) genistein, (**h**) kaempferol, (**i**) myricetin, and (**j**) quercetin. The competitive binding affinity of ERα and ERβ using (**C**) 17β-oestradiol (E2) and aqueous (**D**) LP extract at various concentrations. Figures are selected as representative data from three independent experiments. Results are expressed as mean ± SD values. * *p* < 0.005 (vs. starting concentration, 1.5625 µg/mL).

Molecular docking also suggested that a particular amino acid residue, Arg394 and Glu353, was involved in a common hydrogen bond interaction between the LP phytochemicals and ERα. In contrast, amino acid residues Arg346, Glu305, and L339 were the common hydrogen bond interactions in ERβ (Table 3). According to Figure 1A and Figure 1B, all LP phytochemicals bind to the same site as 17β-oestradiol in both ERα and ERβ. This suggests that they function similarly to phytoestrogens and mimic the effects of oestrogen [27].

### 2.2. Competitive Binding of LP Extracts to the Oestrogen Receptor

The ability of the LP molecule to bind to the ER was evaluated using a TR-FRET oestrogen receptor α and β competitive binding assay based on fluorescence polarisation to assess the oestrogenic activity of the aqueous LP extract. The assay worked by binding a terbium (Tb)-labelled antibody to the oestrogen receptor protein, which then acted as a donor fluorophore. When a fluorescently labelled oestrogen molecule (the acceptor) is bound to the ER’s ligand binding domain, the proximity allows for energy transfer from the Tb to the acceptor, producing a detectable fluorescent signal. When a test compound with an affinity for ER is introduced, it competes with the fluorescent tracer for binding, resulting in a decrease in the TR-FRET signal, which inversely reflects the binding strength of the compound. The assay showed that LP can bind to ERs and mimic the activity of 17β-oestradiol towards ERα and ERβ. The ability of the LP molecule to bind ERα and ERβ was evaluated using a competitive binding assay based on fluorescence polarisation to assess the oestrogenic activity of aqueous LP extract. Figure 1C and Figure 1D shows that LP can bind to ERα and ERβ. E2 showed increased binding affinity to ERα and ERβ with increasing concentration. However, 100 nM of 17β-oestradiol showed higher binding affinity to ERβ than ERα with 0.965 and 0.696 TR-FRET ratios, respectively. LP also exhibited the same pattern as 17β-oestradiol, with the binding affinity of the extract increasing with increasing extract concentration. LP showed more affinity for ERα than for ERβ at each concentration.

### 2.3. Inhibition of MCF-7 Cell Proliferation Using LP Extract

**Figure 2:**
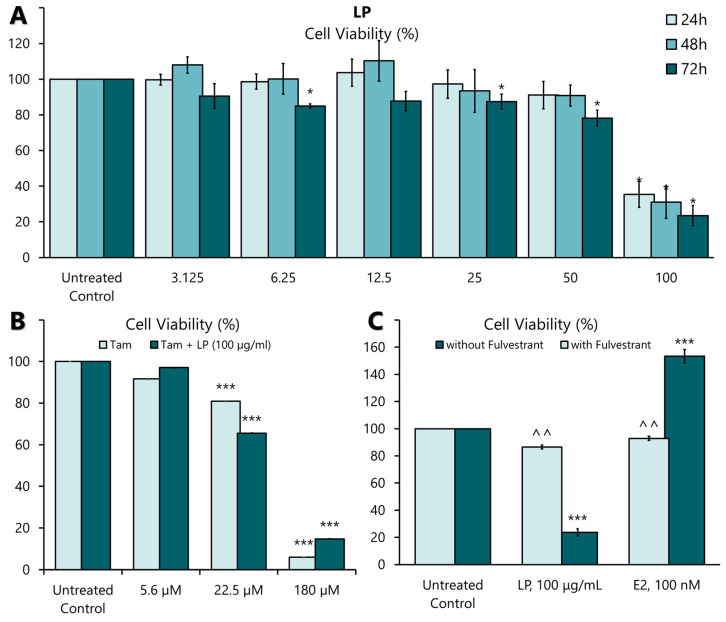
The effect of LP on MCF-7 cell proliferation. (**A**) The effect of aqueous LP extracts on MCF-7 cell proliferation rates after 24, 48, and 72 h of incubation. (**B**) The effect of LP aqueous extract on tamoxifen-treated MCF-7 cells. (**C**) The effect of 17β-oestradiol (E2) and LP extract on MCF-7 cells with and without 100 nM Fulvestrant supplementation. The cell proliferation rate at each concentration was calculated as a percentage of the untreated control. Figures are selected as representative data from three independent experiments. Results are expressed as mean ± SD values. * *p* < 0.005 (vs. untreated control). *** *p* < 0.005 (vs. untreated control), ^^ *p* < 0.005 (vs. without Fulvestrant).

LP induced significant antiproliferative activity in MCF-7 cell lines in a dose- and time-dependent manner (Figure 2A). The highest antiproliferative effect of the extract was observed at 50 and 100 µg/mL at 24, 48, and 72 h. Among the three incubation times, the antiproliferative effect was most pronounced at a concentration of 100 µg/mL after 72 h. The IC_25_, IC_50_, and IC_75_ values are presented in Table 4. The antiproliferative effect of tamoxifen, a positive control on MCF-7, showed a significant antiproliferative effect in MCF-7 cells at 22.5 µM (8.3 mg/mL) and 180 µM (66 mg/mL) concentration (Figure 2B). When the aqueous extract of LP was combined with tamoxifen, the plant extract did not negate the effect of the drug (Figure 2B). These findings show that LP can effectively inhibit the proliferation of MCF-7 cells.

### 2.4. Oestrogen Receptor-Dependent Activity of LP Extracts in MCF-7 Cells

Activation of ERs requires the binding of a ligand, such as 17β-oestradiol (E2). Therefore, the oestrogenic activity of the aqueous extract of LP was investigated by incubating MCF-7 cells with the extract at IC_50_ value (91.19 µg/mL) and E2 (100 nM) in the presence or absence of an oestrogen receptor antagonist, Fulvestrant (ICI 182780) (100 nM), for 24 h. As shown in Figure 2C, the proliferative effect of E2 was significantly blocked in the presence of Fulvestrant. Similarly, the antiproliferative effect of LP was also blocked by Fulvestrant. This result suggests that ERs mediate the oestrogenic action of the aqueous extract of LP.

### 2.5. Flow Cytometry Analysis of the Apoptotic Activity of LP Extracts

**Figure 3:**
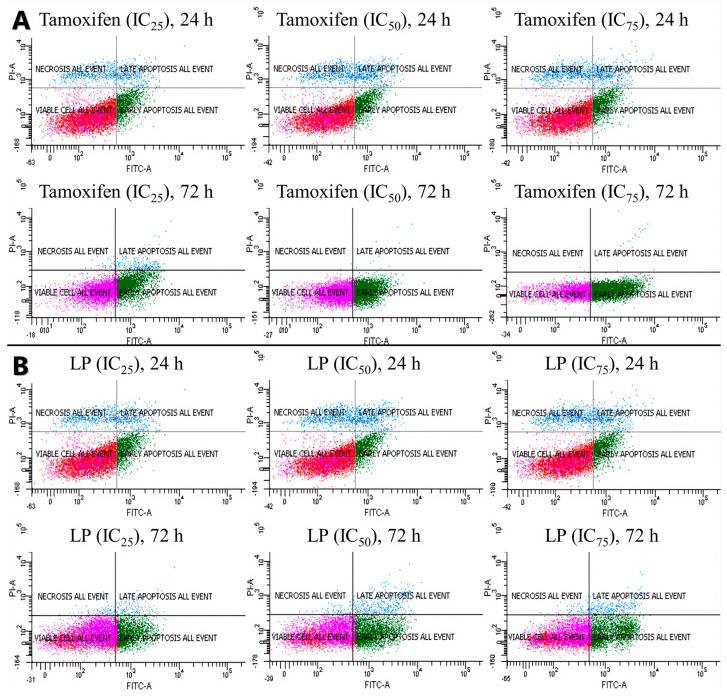
Cells were treated with tamoxifen (**A**) and LP (**B**) at indicated times (24 and 72 h) and concentrations (IC_25_, IC_50_, and IC_75_) and then were co-stained with PI and annexin V-FITC. The translocation of phosphatidylserine was detected using flow cytometry after both tamoxifen and LP treatment.

**Figure 4:**
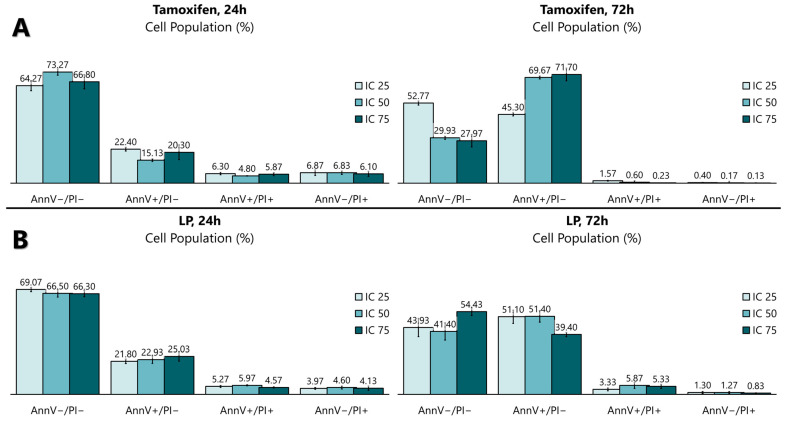
Quantitative analysis of the apoptosis flow cytometry analysis using (**A**) tamoxifen- and (**B**) LP-treated MCF-7 cells. The graph was selected as representative data from three independent experiments. Results were expressed as mean ± SD values.

The antiproliferative activity of the LP extract was determined using the FITC Annexin V apoptosis detection kit (Figure 3 and Figure 4) to determine the cell death of MCF-7. The flow cytometry analysis showed that, from 24 to 72 h of incubation with tamoxifen, the early apoptotic cell population increased from 15.1% to 69.7%, while the viable cell population decreased from 73.2% to 29.9%. Like tamoxifen, LP aqueous extracts also induced a significant level of apoptosis in MCF-7 cells. The early apoptotic cell population at 24 h increased from 22.9% to 51.4% after 72 h, while the viable cell population decreased from 66.5% (24 h) to 41.4% (72 h).

### 2.6. Expression of Apoptotic Markers in MCF-7 Cells by LP Extracts

**Figure 5:**
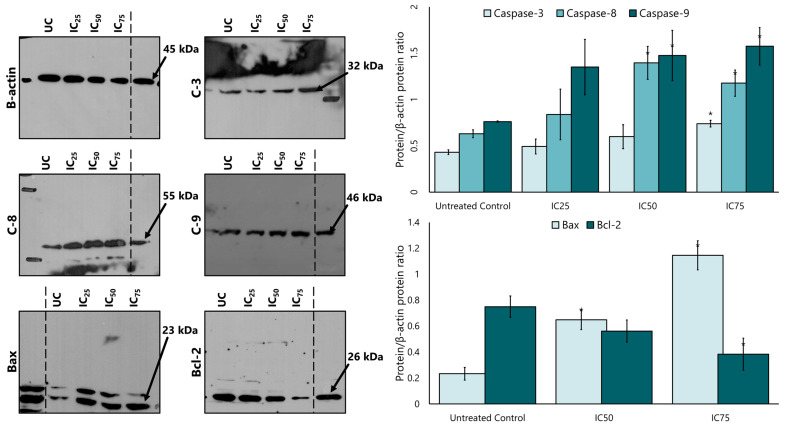
The expression of caspases 3, 8, and 9, and the intrinsic apoptotic proteins Bax and Bcl-2 in MCF-7 cells after treatment with various concentrations of the aqueous extract of LP. SDS-PAGE and Western blotting evaluated protein expression. Densitometry for all proteins was calculated as the ratio to β-actin. UC = untreated control. Figures are selected as representative data from three independent experiments. Results are expressed as mean ± SD values. * *p <* 0.01 (vs. untreated control).

Caspase 8, caspase 9, and caspase 3 proteins were analysed to determine whether the LP extract promotes apoptosis via a caspase-dependent pathway. Western blotting findings showed that LP treatment increased the expression of caspase 8, caspase 9, and caspase 3 protein in a dose-dependent manner (Figure 5). Quantitative densitometry analysis showed a higher expression of caspase 8, 9, and 3 in MCF-7 cells after treatment with LP compared to the untreated control cells. Caspase 9 showed the highest expression among the three caspases at all IC_25_, IC_50_, and IC_75_ concentrations, followed by caspase 8 and caspase 3.

After the increased expression of the intrinsic apoptotic protein caspase 9, the study continued with the expression of the proteins Bcl-2 and Bax. Figure 5 also shows that the expression of Bax increased in a dose-dependent manner compared to the untreated control, while the expression of Bcl-2 also decreased after treatment with LP extract.

## 3. Discussion

LP has been documented by traditional Eastern medicine, especially as Malay traditional medicine, to treat menopausal syndrome [33]. LP is also known for its oestrogenic properties [33,34,35,36,37]. The main use of this is to balance oestrogen hormones in women. Currently, extensive research has been conducted to investigate the phytochemicals of LP, indicating the significant potential of the plant to treat various human health conditions [38]. There is ample evidence for LP’s oestrogenic effect. A previous study using the same LP extract as in this study showed that many bioactive compounds, including four alkyl resorcinol derivatives and one dimeric 1,4-benzoquinone, exhibited selective binding affinities towards both the ERα and ERβ subtypes [32,33]. In addition, a review article from 2013 found that LP contains various phytochemicals, including phenolic acids and flavonoids, which have beneficial biological properties [21]. However, there is a paucity of information on its antiproliferative properties. In this study, we investigate the relationship between LP’s oestrogenic properties and its antiproliferative action and the possible mechanism of its anticancer effect. This study is the first attempt to explore the mechanism underlying the impact of the anticancer activity of LP aqueous extract on breast cancer cells.

The phytoestrogens found in LP can bind to the oestrogen receptors ERα and ERβ at the same site as 17β-oestradiol. Of the phytoestrogens, daidzein exhibited the lowest binding affinity, but formed hydrogen bonds most similar to those of 17β-oestradiol, particularly with ERα. These docking results indicated a strong binding affinity between some of the phytoestrogens, especially daidzein and genistein, in LP at both oestrogen receptors, suggesting that the protein was in a favourable conformation. Based on the results of molecular docking, the ER binding assay was performed and showed that the aqueous extract of LP had a substantial oestrogen-like activity (1.5625–100 µg/mL). Similar results were obtained in various studies with plant extracts known to contain these phytoestrogens. For example, water extracts of *Adenophora triphylla* and *Agrimonia pilosa* were able to bind to oestrogen receptors and repress the binding of E2 to ERα and Erβ [38,39]. These results are further evidence in favour of the oestrogenic activity of LP. Consequently, LP phytochemicals may serve as potential alternatives for hormone replacement therapy in postmenopausal women.

The cytotoxicity of the aqueous LP extract using the MTT assay initially showed that the extract inhibited the proliferation of MCF-7 breast cancer cells in a dose- and time-dependent manner, especially at 50 and 100 µg/mL in all three incubation periods. This finding is consistent with previous studies in which cytotoxicity increased with increasing concentration of the extract [36,40]. Study by Karimi et al. in 2016 demonstrated that the CO_2_-enriched plant extract showed a cytotoxic effect on breast cancer cells [41].

Interestingly, low concentrations (3.125, 6.23, 12.5, and 25 µg/mL) of LP aqueous extract slightly increased the proliferation of MCF-7 cells compared to the untreated control. These findings support a previous study by Zheng et al., showing that genistein has a biphasic effect on MCF-7 cells, a potent agonist and cytotoxic in a concentration range of 10 nM–20 μM [42]. The biphasic effect occurs when a compound has an opposite effect at low and high concentrations. In this study, LP extract showed an antiproliferative effect at high concentrations (>50 μg/mL), while at low concentrations (<50 μg/mL), it showed an oestrogenic effect on MCF-7 cells. However, the proliferation rate of MCF-7 cells at low concentrations (<50 μg/mL) of LP extract did not show any significant result. When MCF-7 cells were incubated with Fulvestrant (ICI 182,780), a known ER antagonist, the antiproliferative effect of the LP extract was blocked, indicating that the oestrogen-receptor-mediated antiproliferative effect of LP extract is blocked in MCF-7 cells. These results suggest an oestrogen-receptor-related mechanism for the anticancer effect on MCF-7 breast cancer cells.

Cell death can be studied using various methods, including tunnel assays, PCR, and flow cytometry analyses. In this study, apoptotic cell death was analysed using flow cytometry tagged with Annexin V and PI. Cells undergoing the apoptotic process exhibit different morphological states. In early apoptotic cells, for example, the phospholipid phosphatidylserine membrane is translocated to the outside of the plasma membrane, exposing the external cellular environment [43,44]. This allows for Annexin V to enter the inner membrane of the cell and bind to the PS. Normal cells with intact membranes will reject PI, while dead or damaged cells are permeable to PI. Therefore, viable cells are Annexin V and PI negative, early apoptotic cells are Annexin V positive and PI negative, and late apoptotic cells (or necrosis cells) are Annexin V and PI positive. Interestingly, the results show that the aqueous extract of LP did not cause a significant difference between the concentrations of LP, but significantly increased the percentage of MCF-7 cells in the early apoptotic stage in a time-dependent manner. Therefore, the antiproliferative effect of LP in MCF-7 cells is thought to be through the induction of apoptosis, which is consistent with tamoxifen and the data from the previous MTT assay. In addition to MCF-7 breast cancer cells, other cancer cells like melanoma, prostate cancer, hepatocellular carcinoma, and colon cancer also significantly decreased their proliferation rate after the induction of LP. However, none of the above studies specify what types of cell death, apoptosis, or necrosis occur after LP treatment.

Apoptosis is mediated by two different pathways, the intrinsic mitochondria-dependent pathway and the extrinsic death receptor pathway, which is, however, controlled by the same members of the cysteine protease family of an enzyme called caspase [43]. The activation of caspases can be seen as an indication of the apoptotic mode of cell death [44,45]. Caspase 9 activates the intrinsic pathway, while caspase 8 activates the extrinsic pathway. Pro-caspase-3, an inactive form of caspase 3, were then cleaved by caspase-8 or caspase-9. This leads to the activation of caspase 3, where the onset of apoptosis occurs. In this study, the expression of caspase 3, caspase 8, and caspase 9 was elevated after cells were treated with an aqueous extract of LP. Caspase 8 is an initiator caspase involved in death-receptor-triggered apoptotic signalling, i.e., the extrinsic pathways [46]. A previous study has shown that caspase 8 can be activated not only by the death receptor, but also independently by direct cleavage through other proteases, such as granzyme B [47]. A study by Chung et al. found that caspase 8 is activated in HeLa cells by the flavonoid eupafolin without the involvement of a death receptor [45]. The study also showed that caspase 8 is activated after the activation of caspase 3 and 9. Therefore, it was hypothesised that caspase 3 is required for the activation of caspase 8 during eupafolin-induced apoptosis. Caspase 3 is the primary execution caspase responsible for PARP cleavage, which causes the biochemical and morphological changes observed in apoptotic cells [48]. This could explain why the expression of caspase 3 in this study is lower than that of caspase 8 and caspase 9.

In addition to the caspase proteins, Bax and Bcl-2 are also important for apoptosis. Bax and Bcl-2, pro- and anti-apoptotic proteins, come from the Bcl-2 family, which is activated by the Bid protein. The ratio between these two proteins determines whether the cell will undergo apoptosis. Activation of Bax leads to an intrinsic apoptotic pathway by releasing cytochrome C from the mitochondria into the plasma membrane and increasing PARP cleavage. A study by Pihie et al. demonstrated that LP triggers intrinsic apoptosis cell death using an in vitro assay. The study concluded that the active fraction of LP, SF2Lp, induced apoptosis through up- and down-regulation of the Bax/Bcl-2 protein mediated by a p53-dependent pathway [47].

In this study, the expression of Bcl-2 was significantly reduced, and that of Bax increased in MCF-7 cells treated with LP aqueous extract. The changes in the levels of these two proteins are consistent with the state of apoptosis in cells treated with MCF-7 [49,50]. The balance between the Bcl-2 family proteins, anti-apoptotic and proapoptotic, may be associated with an increase in mitochondrial outer membrane permeabilisation (MOMP), leading to the intrinsic apoptotic pathway [48].

**Figure 6:**
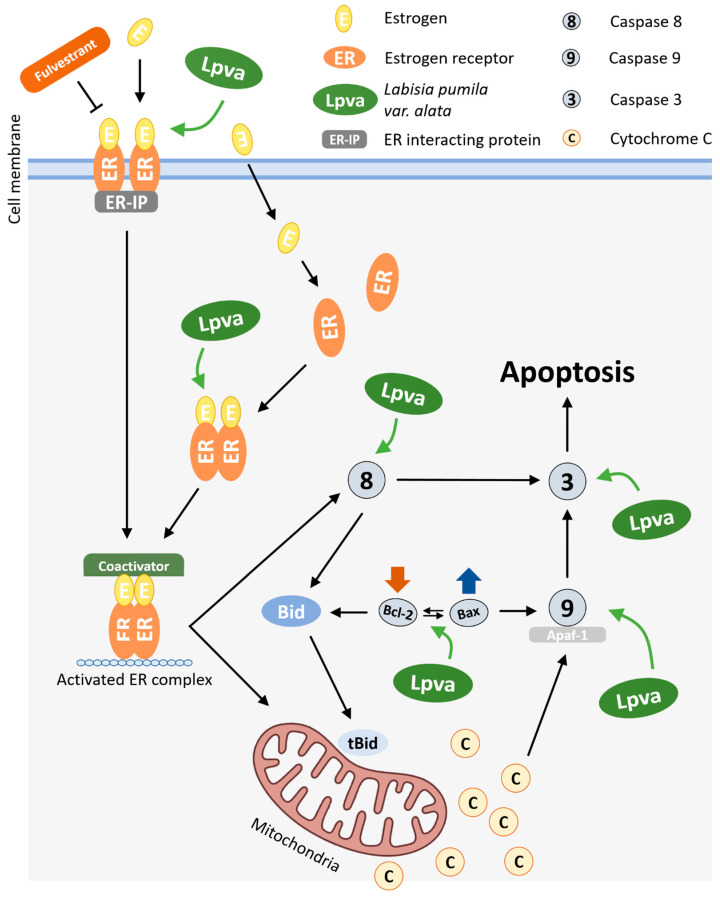
Possible molecular mechanism of action of LP on intrinsic and extrinsic apoptosis pathways in MCF-7 cells.

Based on the findings of this study, the possible mechanism by which LP exerts an antiproliferative activity toward MCF-7 cell lines is presented in Figure 6. In a previous study, LP was found to contain bioactive phytochemical compounds, including phenolics, flavonoids, and other antioxidants such as alkenyl, saponins, and benzoquinone derivatives [21]. These bioactive compounds from LP have many biological activities, such as antioxidant properties, as well as anti-inflammatory, anti-ageing, antimicrobial, and antifungal activities. It is believed that the anticancer properties of this plant are due to its numerous phytoestrogens. Moreover, depending on the ER subtype (α or β) present in the cells, these phytoestrogens, which mimic the role of oestrogen, can both support cell survival and trigger cell death by apoptosis [46]. It would be interesting to investigate the role of LP in the different types of oestrogen receptors. In addition, more in-depth experiments are needed to explore the mechanisms by which bioactive compounds, as an isolated compound, are associated with LP-induced cell death in MCF-7 cells.

The antiproliferative and apoptotic effects observed in MCF-7 cells following treatment with LP are probably due to its multiple bioactive compounds, including phenolics, flavonoids, saponins, and benzoquinone derivatives. Phytoestrogens in particular are of special interest due to their ability to interact with oestrogen receptors (ERα and ERβ). This interaction can lead to either cell survival or apoptosis, depending on which receptor subtype is expressed. This suggests a possible mechanism by which LP exerts its anticancer effects and emphasises the need for further investigation of its receptor-specific interactions.

These findings are consistent with existing studies on the chemopreventive and chemosensitising properties of dietary bioactive compounds in breast cancer [12]. They support the notion that such compounds may contribute significantly to the therapeutic potential of plant-based remedies and diets. Further research into the isolated compounds in LP could clarify their individual or synergistic roles, providing valuable insights for the development of plant-derived anticancer strategies.

## 4. Materials and Methods

### 4.1. Materials

Roswell Park Memorial Institute (RPMI) 1640 medium, phenol red-free RPMI, foetal bovine serum (FBS), and charcoal dextran-coated were purchased from Gibco (Grand Island, NY, USA). Tamoxifen, 17β-oestradiol, ICI 182780 (Fulvestrant), and MTT solution were purchased from Sigma-Aldrich (St. Louis, MO, USA). Proapoptotic proteins such as caspase 9, caspase 8, caspase 3, Bad, β-actin, and their secondary antibodies for each protein were purchased from Santa Cruz Biotechnology (Santa Cruz, CA, USA). Bcl-2 was purchased from Cell Signaling Technology (Danvers, MA, USA).

### 4.2. Sample Preparation

The dried leaves of *Labisia pumila* var. *alata* (LP) were provided by Bioalpha Sdn. Bhd, and a voucher specimen (FF/UiTM/KF/02/13) is deposited in the Faculty of Pharmacy, Universiti Teknologi MARA. The dried component was extracted with water at a ratio of 1:10 for 2 h at 100 °C. Then, the extract was concentrated at 55 °C for 4 h before being filtered and spray-dried. Approximately 10 mg of the dried LP powder was suspended in double-distilled water and sterile-filtered to produce a stock solution of 10 mg/mL. The stock samples were stored at −20 °C for further analysis.

### 4.3. Molecular Docking Analysis and Simulations

Docking analysis was performed using AutoDock Vina software version 1.2.0 to determine the affinity and site of binding of the target ligand to ER. The 3D structures of ERα (5WGD) and ERβ (5TOA), of which both already contain ligand 17β-oestradiol, were downloaded in PDB format from the RSCB Protein Data Bank website. The ligand was extracted from the receptor, and the active site was then validated by redocking the original co-crystallised 17β-oestradiol back into the protein. The target compound used in this analysis was the phytochemicals found in LP, which were summarised in a previous publication by Abdullah et al. from 2013 [21]. Daidzein (CID 5281708), genistein (5280961), apigenin (5280704), quercetin (5280343), kaempferol (5280863), catechin (9064), myricetin (5281672), epigallocatechin (72277), caffeic acid (689043), and gallic acid (370) were downloaded from PubChem library in SDF format and later converted to PDB format. In addition, 17β-oestradiol (5757) was used as a positive control.

All ligands were prepared by defining the torsion tree for each phytochemical, and the format was converted to pdbqt format using AutoDock Tools. Both ERα and ERβ were prepared by removing water molecules, replacing them with polar hydrogen, and converting the format to pdbqt format with a suitable grid box size (36 × 36 × 36 Angstrom (Å)). The centre of location depends on the active site of each receptor. All docking analyses were performed three times in triplicate.

### 4.4. Oestrogen Receptor Binding Assay

The ER binding ability of LP water extract at various concentrations was performed using the TR-FRET ERα competitive binding assay and the TR-FRET ERβ competitive binding assay from Invitrogen (Waltham, MA, USA). The procedure was performed according to the manufacturer’s instructions. The concentrations of 100, 50, 25, 12.5, 6.25, 3.125, and 1.5625 µg/mL aqueous extract of LP were used based on the manufacturer’s recommendation and a previous study with slight modifications [38,39]. The ratio of TR-FRET was calculated by dividing the value of 520 nm by the 495 nm emission signal. 17β-oestradiol (E2) was used as a positive control.

### 4.5. Cell Culture and Maintenance

ER-positive cell lines, MCF-7 breast adenocarcinoma cells, were purchased from the American Tissue Culture Collection (Mannasas, VA, USA). Cells were cultured with RPMI medium supplemented with 10% FBS, 1% 100× penicillin/streptomycin, and 0.5% gentamicin (Gibco, Grand Island, NY, USA) in a humidified atmosphere of 37 °C in an incubator of 5% CO_2_. The medium was renewed 2–3 times per week, and the subculture was initiated when cell confluence reached 80–90%. Since MCF-7 is an oestrogen-sensitive cell, both RPMI and FBS were replaced with phenol red-free RPMI with 5% charcoal-dextran stripped FBS prior to treatment incubation with LP extracts to minimise its oestrogenic effect, as phenol red has been shown to have oestrogenic properties in hormone-sensitive cells such as MCF-7 cell lines [51]. Together with charcoal dextran-stripped FBS, this study ensured that the effect on MCF-7 cells was exclusively due to the aqueous LP extracts.

### 4.6. MTT Assay

An MTT assay was used to evaluate the antiproliferative effect of LP extract. Yellow tetrazolium (3-(4, 5-dimethylthiazolyl-2)-2, 5-diphenyltetrazolium bromide) is reduced to purple formazan by mitochondrial dehydrogenase in viable cells [52]. MCF-7 cells were seeded in a 96-well plate at different cell densities (20,000, 10,000, and 8000 cells per well) for three different incubation times (24, 48, and 72 h). After overnight incubation, the medium was replaced with varying concentrations of LP extract (100, 50, 25, 12.5, 6.25, 3.125 µg/mL) in phenol red-free RPMI with 5% charcoal-dextran stripped FBS. Untreated cells served as negative control, while tamoxifen and 17β-oestradiol (Sigma, St. Louis, MO, USA) served as positive controls, depending on the assay. Furthermore, the LP-treated cells were added with or without Fulvestrant (ICI 182,780) (Sigma, St. Louis, MO, USA). After incubation, 50 µL of a 5 mg/mL MTT solution was added to each well and incubated in the dark at 37 °C for a further 4 h. The medium in each well was then replaced with 200 µL of a 100% filtered DMSO solution and placed on a plate shaker for 5 min. The absorbance was measured using a microplate reader at 540 nm.

### 4.7. Flow Cytometry Apoptosis Analysis

Flow cytometry analysis was performed using the FITC Annexin V apoptosis kit (Invitrogen, Carlsbad, CA, USA) according to the manufacturer’s instructions [53]. MCF-7 cells were seeded in a 6-well plate at a density of 2 × 10^6^ cells/well and incubated for 24 h in phenol red-free medium and 5% charcoal-dextran stripped FBS at 37 °C and 5% CO_2_. After incubation, the cells were treated for 24 and 72 h with the values IC_25_ (105.48 µg/mL), IC_50_ (91.19 µg/mL)_,_ and IC_75_ (71.16 µg/mL) of the LP extract determined from the MTT assay, with tamoxifen serving as a positive control. The concentration of tamoxifen used in this experiment was based on a previous study with slight modifications [54]. After treatment incubation, cells were detached using 0.05% Accutase from Thermo Fisher Inc. (Waltham, MA, USA) and resuspended in 400 µL of 1× Annexin binding buffer. Then, 5 µL of annexin V and 5 µL propidium iodide were added and further incubated for 15 min in the dark. The cells were then analysed using the FACS Canto II flow cytometry machine by BD Biosciences (Franklin Lakes, NJ, USA).

### 4.8. Western Blot Analysis

Protein expression of the apoptotic markers, caspase 3, caspase 8, caspase 9, Bax, and Bcl-2, was quantified using the Western blot technique [44]. MCF-7 cells were seeded in a 6-well plate at a density of 20,000 cells/well and incubated for 24 h. The LP extracts were treated in three different concentrations: IC_25_ (105.48 µg/mL), IC_50_ (91.19 µg/mL), and IC_75_ (71.16 µg/mL) for 16 h. The untreated well was considered as a negative control. The preparation of cell lysates as sample loading and Western blot analysis was performed according to the procedures described by Ismail et al, with slight modifications [55,56].

The MCF-7 cell lysate was prepared by mixing with 100 µL lysis buffer (NP40, HEPES, glycerol, and NaCl). The lysate was collected, incubated on ice for 10 min, and then centrifuged at 10,000 rpm at 4 °C. The supernatant was transferred to a microcentrifuge tube, mixed with laemli buffer (5:1), and heated at 100 °C for 5 min. The lysate can be used directly or stored at −80 °C for the next step.

After that, the lysate was loaded and separated on SDS-PAGE gel electrophoresis at 150 V and 60 A for 1 h. Following gel electrophoresis, the separated protein mixtures were transferred to a nitrocellulose membrane filter paper from GE Healthcare (Chicago, IL, USA) for further analysis. The membrane was destained with deionised water and was assembled according to the Merck SNAP ID Protein Detection System protocol. Then, the membrane was blocked with 5% skimmed milk in a TBST mixture (0.05 M Tris-HCl, Tris base, 0.15 M NaCl, and 0.1% Tween 20) for 10 min. The membrane was then incubated with a specific primary antibody (caspase 3, caspase 8, caspase 9, Bax, Bcl-2, and β-actin) at a ratio of 1:1000 for 10 min with the same milk and TBST mixture and washed four times with 30 mL Wash Buffer (Chemiluminescence Substrate Kit, KPL). The membrane was then incubated with a secondary antibody using the same procedure as the primary antibody.

The membrane was enhanced using a chemiluminescent dye (Thermo Fisher Scientific) before being transferred to X-ray film. The band intensity that appeared from the film was evaluated using MyImageAnalysis software (Thermo Scientific) version 2.0 to quantify the chemiluminescent intensity and normalised with β-actin protein as a loading control.

### 4.9. Statistical Analysis

Data in this study were expressed as mean ± standard deviation (SD) for three different tests (*n* = 3). The result was processed using one-way analysis of variance (ANOVA) using Sigma Plot version 12 software. Statistically different means were recognised at *p* < 0.05.

## 5. Conclusions

In conclusion, the current study confirms that the aqueous extract of LP induces an antiproliferative effect through the activation of the ERα and the ERβ followed by caspase-dependent apoptosis via intrinsic and extrinsic pathways, which increases the expression of caspase 9 and caspase 8 and alters the ratio of Bcl-2/Bax protein in MCF-7 cells. The elucidation of the mechanism of LP, which is mediated between the oestrogen and apoptosis signalling pathways, may offer an opportunity to develop new therapeutic strategies for the treatment of breast cancer.

## Figures and Tables

**Table 1:** *Labisia pumila* compositions [20,21,22,32].

Compounds			
Phenolics	Gallic Acid	Flavonoids	Quercetin
	Caffeic acid		Myricetin
	Pyrogallol		Epigallocatechin
	Benzoic acid		Catechin
	Cinnamic acid		Naringin
	Methyl gallate		Rutin
Saponins	Ardisicrenoside B		Apigenin
	Ardisiacrispin A		Daidzein
	Cyclamiretin A		Genistein
	Ardisimamilloside H		Beta carotene
Alkenyl and benzoquinone derivatives			Ascorbic acid

**Table 2:** The binding affinity of 17β-oestradiol and LP phytochemicals towards ERα and ERβ.

Ligands	Estimated Binding Affinity (kcal/mol)	
	Oestrogen Receptor α	Oestrogen Receptor β
17β-oestradiol (Control)	−9.6	−11.2
Daidzein	−9.1	−9.1
Genistein	−9.0	−9.1
Apigenin	−8.8	−8.8
Quercetin	−8.5	−8.8
Kaempferol	−8.4	−8.4
Catechin	−8.3	−8.3
Myricetin	−8.2	−8.1
Epigallocatechin	−8.1	−7.8
Caffeic Acid	−6.3	−6.6
Gallic Acid	−5.6	−5.9

**Table 3:** Hydrogen bond interactions with 17β-oestradiol and LP phytochemicals towards ERα and ERβ.

Ligands	Hydrogen Bond Interactions and Their Bond Length	
	Oestrogen Receptor α	Oestrogen Receptor β
17β-oestradiol (Control)	Glu353 (2.9), Leu387 (3.3), His524 (3.3), Arg394 (3.4), Gly521 (3.6)	Glu305 (2.0), His475 (3.0), Arg346 (3.4)
Apigenin	Leu387 (2.5), Glu353 (2.6), Arg394 (3.3)	Leu339 (2.0), Arg346 (3.0), Glu305 (3.5)
Caffeic Acid	Glu353 (2.0), Leu387 (2.3), Arg394 (3.0)	Leu339 (2.2), Arg346 (3.2)
Catechin	Glu353 (1.9, 2.7), Arg394 (3.4)	Leu339 (2.1), Glu305 (2.3), Arg346 (3.3)
Daidzein	Glu353 (2.0), His524 (2.3), Leu387 (2.5), Arg394 (3.3)	His475 (2.6), Glu305 (2.7), Arg346 (2.8)
Epigallocatechin	Glu353 (1.9), Leu387 (2.1), His524 (2.2)	Glu305 (1.6), Leu339 (2.6), Glu305 (1.6), Leu339 (2.6), Arg346 (3.4), His475 (2.7)
Gallic Acid	Leu387 (2.5)	Glu305 (1.8)
Genistein	Glu353 (1.9), Arg394 (2.2), His524 (2.2)	His475 (2.1), Glu305 (2.3), Arg346 (2.8)
Kaempferol	Arg394 (3.0)	Leu339 (2.1), Gly472 (2.2), Arg346 (3.0)
Myricetin	Glu353 (2.0), Leu387 (2.1), Arg394 (3.3)	Leu339 (2.1), Glu305 (2.4)
Quercetin	His524 (1.8), Leu387 (2.0), Glu353 (2.5), Arg394 (3.0,3.1)	Glu305 (2.0), Leu339 (2.2), His475 (2.4), Arg346 (3.2)

**Table 4:** The IC values of LP after 24, 48, and 72 h incubation on MCF-7 cell lines.

Incubation Period	LP Aqueous Extract IC Value (µg/mL)		
	IC_25_	IC_50_	IC_75_
24 h	105.48	91.19	71.16
48 h	103.88	87.19	65.64
72 h	97.10	71.65	53.23

## Data Availability

Data are contained within the article.
